# Eczema Herpeticum Complicating Atopic Dermatitis: A Rare Presentation

**DOI:** 10.7759/cureus.55171

**Published:** 2024-02-28

**Authors:** Mariana Pedro, Marta Caldas, Fernanda Neves, Sara Diogo, Fabiana Fortunato

**Affiliations:** 1 Pediatrics, Centro Hospitalar do Oeste, Unidade Caldas da Rainha, Caldas da Rainha, PRT; 2 Dermatology, Centro Hospitalar do Oeste, Unidade Caldas da Rainha, Caldas da Rainha, PRT

**Keywords:** pediatrics, dermatology emergency, simplex herpes virus, eczema herpeticum, atopic dermatitis

## Abstract

Atopic dermatitis (AD) has become a global health concern due to an increase in its frequency over the past few decades. This illness not only reduces the quality of life but also imposes a considerable financial burden due to the increased risk of skin infections.

This case report explores the presentation of a four-month-old male infant with a personal history of atopic dermatitis that developed yellow scaly lesions on the scalp, which were assumed to be cradle cap. However, there was a clinical worsening of the cutaneous lesions, with the appearance of vesicles, so he was referred to the Pediatric Emergency Room after an urgent dermatology appointment. A blood test was performed, which revealed severe eosinophilia and a slightly increased total IgE. Considering the patient's past medical record of atopic dermatitis and the observable characteristics of the skin rash, there was a strong suspicion of eczema herpeticum (EH). Consequently, intravenous acyclovir treatment was initiated, along with an antibiotic, as there were concerns about a potential secondary infection. He was followed up with a pediatric and dermatology appointment, with a resolution of skin lesions after six weeks.

EH is a rare clinical entity, usually caused by herpes simplex virus (HSV) types 1 and 2. It is a clinical entity that, while being uncommon, is one of the few dermatological emergencies responsible for a high morbidity rate, associated with the systemic spread of the viral infection.

## Introduction

Atopic dermatitis (AD) is a chronic and relapsing inflammatory skin disease with an increasing incidence, especially in developed countries [1]. It is the most common chronic inflammatory skin disease, with a prevalence of 15%-25% in children [1-3]. In most cases, symptoms appear before the age of five; however, AD can occur throughout life. Moreover, most children with AD will outgrow the disease, but 25% of children will continue to have symptoms into adulthood [4].

AD patients are more likely to develop skin infections. Moreover, it is important to highlight that Staphylococcus aureus infections are particularly prevalent in AD patients and can result in superinfection. Regarding viral infections, patients with AD might have widespread clinical rashes due to the herpes simplex virus (HSV), molluscum contagiosum virus (MCV), human papillomavirus (HPV), coxsackievirus, and vaccinia virus (VV) infections. Eczema herpeticum (EH), eczema molluscatum, eczema verrucatum (EV), eczema coxsackium (EC), and eczema vaccinatum are the nomenclature of the rash types that correlate with the virus that causes them [5]. When it comes to viral infections, the common clinical rashes caused by HSV, MCV, HPV, coxsackievirus, and VV are mostly harmless. However, a significant life-threatening condition can result from EH and EV [6,7].

EH was first described in 1887 by Moritz Kaposi and can be categorized based on how severe it is: from local to widespread infection, sometimes leading to herpetic encephalitis. HSV-1 is the root cause of EH in more than 90% of patients. Furthermore, in addition to the primary infection, the reactivation of the latent virus is linked to this condition. Despite the fact that many people are HSV-seropositive, EH only rarely affects AD patients. Regarding EH prevalence, it is a relatively uncommon condition, and it is predicted that 3% of AD patients will experience EH during their lives [2]. Although EH can impact individuals of any gender, a prior investigation revealed that the disease tends to manifest more severely in male patients [8]. The HSV, comprising both HSV type 1 (HSV-1) and type 2 (HSV-2), is a double-stranded DNA virus enclosed in a structure. It belongs to the Herpesviridae family and the Alphaherpesvirinae subfamily. Human exposure to these infections is common, with approximately 80% seroprevalence for HSV-1 in children and 90% in adults [4,9,10]. While HSV-1 typically leads to labial, ophthalmic, and primary genital herpes, HSV-2 is primarily associated with sexually transmitted genital herpes [11]. This article presents a representative clinical case, offering valuable insights into this rare manifestation within the realm of dermatology.

## Case presentation

We present the case of a four-month-old male infant with a personal history of AD who was apparently well until two weeks before being admitted to the hospital. At that time, yellow scaly lesions started to emerge on his scalp, and cradle cap was assumed. Due to concerns that skin lesions might have worsened, he was reexamined at the primary care unit after one week. During the physical examination, pruritic, erythematous vesicles with erosion and crusting were observed on his scalp. Consequently, he was promptly referred for an urgent dermatological evaluation and started on a treatment regimen involving amoxicillin and clavulanic acid.

**Figure 1:**
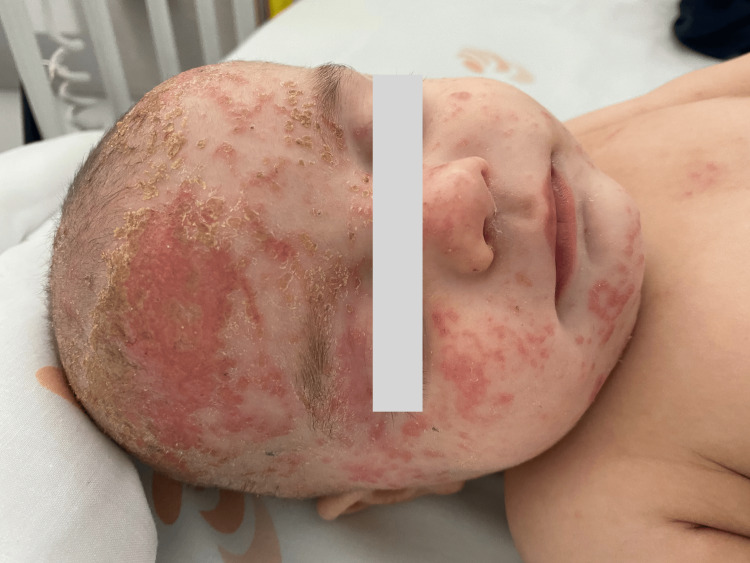
Erythematous vesicles and papules associated with erosion and crusting on the face, scalp, trunk, limbs, and perineum.

Dermatological evaluation showed a spread of the lesions to the face and limbs, although the child still had a healthy appearance. The infant was promptly sent to the Pediatric Emergency Room (PER) because topical therapy was ineffective due to the exuberance of the lesions. On physical examination, he had a good appearance but was feverish and presented with erythematous vesicles and papules associated with erosion and crusting on the face, scalp, trunk, limbs, and perineum (Figure 1).

He had a family member at the time who had active lesions from a herpetic lip infection. A blood test was performed, including HIV serology, immunoglobulin assay, and lymphocyte populations, only to report severe eosinophilia and a slightly increased total IgE. Owing to technical constraints, conducting PCR screening or a direct fluorescent antibody test for HSV on a sample collected from skin lesions was not attainable.

**Figure 2:**
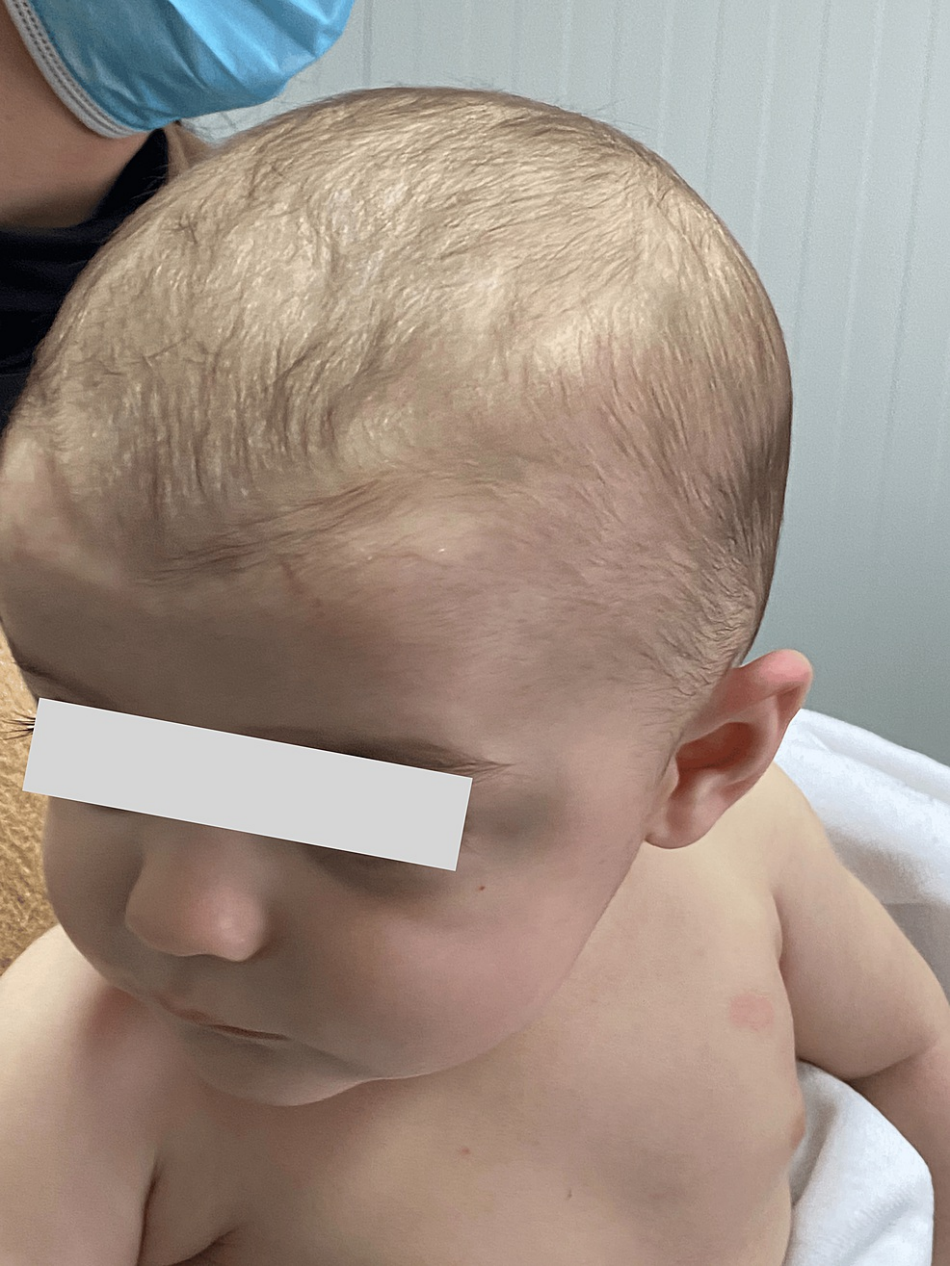
Well-appearing infant after six weeks with complete resolution of skin lesions.

Based on the patient's past medical record documenting AD and the observed clinical characteristics of the skin rash, EH was highly suspected; so, intravenous acyclovir was started right away, coupled with an antibiotic, due to the suspicion of superinfection. The infant's condition significantly improved, and five days later, he was discharged from the hospital with instructions to continue acyclovir and flucloxacillin for an additional five days. He was followed up with a pediatric and dermatology appointment, with a resolution of skin lesions after six weeks (Figure 2).

## Discussion

EH, also recognized as Kaposi’s varicelliform eruption, presents a dermatological emergency with potential life-threatening complications. The initial manifestation of the condition predominantly occurs in children, while the recurrent form is more prevalent among older individuals. Despite AD being the most prominent risk factor, EH may coexist with various other skin conditions, including but not limited to ichthyosis vulgaris, bullous pemphigoid, dyskeratosis follicularis, mycosis fungoides, and contact dermatitis [12]. The compromised skin barrier observed in AD could potentially facilitate virus entry. Moreover, the prevalence of TH2 cells results in the secretion of IL-4, stimulating IgE production while inhibiting the differentiation of IFN-γ-producing TH1 cells. Consequently, the decreased levels of IFN-γ within the skin of AD patients may create conditions conducive to viral proliferation [4].

In EH, even though the hands, legs, or sexual organs may also be affected, the face, neck, and trunk are traditionally the areas that EH most commonly affects. Characteristic manifestations of EH consist of monomorphic eruptions of dome-shaped papulovesicles, and after a week, there is a possibility of observing ulcerations or slits [2,3]. Usually, it takes two to six weeks for lesions to fully dry out and heal [2]. It is sometimes required to consider a potential secondary infection of vesicles by the bacteria *Staphylococcus aureus*, one of the other major complications of EH, when pustules are present [2]. EH is frequently accompanied by fever, malaise, or lymphadenopathy in addition to skin lesions [2,3].

Diagnosis of EH is mainly clinical; however, a polymerase chain reaction (PCR) for HSV-1 and HSV-2 is advised to confirm the diagnosis [2,3]. Aside from performing a molecular test, the following laboratory tests are also possible: Tzanck test, cytological investigation of ulcer content; virological examination of cell culture; and serological examination [13]. This case report emphasizes the need for considering EH as a possible differential diagnosis during the evaluation of patients with exacerbation of AD, particularly when there is an impairment of general health. In this case, the diagnosis was challenging due to the early age of onset, below the first year of life, which is a rare circumstance. Thus, the identification of EH relied solely on clinical observations, substantiated by the rapid clinical enhancement observed with acyclovir treatment. It is crucial to initiate management promptly, as delayed administration of acyclovir has been associated with an elevated likelihood of hospitalization [14]. Moreover, elevated total serum IgE levels and circulating total eosinophil counts serve as risk factors for this condition [3], both of which were evident in this patient.

The gold standard treatment for this illness is acyclovir, administered intravenously for 7 days at a dose of 5-10 mg/kg [2,3]. Furthermore, the mortality rates for untreated EH can be as high as 6-10% [2]. In the context of EH in AD patients, primary prevention would include preventing the primary HSV infection. Immunization of AD patients against HSV is one method that might be used to accomplish it [2,3]. EH can cause considerable morbidity and mortality if diagnosis and treatment are delayed. Before the introduction of acyclovir, the mortality rate associated with EH stood at 50% [2,15]. Furthermore, complications of this condition can include meningoencephalitis, keratoconjunctivitis, and disseminated intravascular coagulation [12]. Given the significant risk of ocular disease problems, ophthalmology observation should be considered, especially if there are periocular lesions or ocular symptoms [16].

## Conclusions

Numerous complications arising from recurrent bacterial and viral skin infections are linked to AD, among which is EH. Therefore, it is crucial for children with active AD to avoid contact with individuals exhibiting active herpes simplex lesions to prevent the onset of this condition. A thorough medical history, considering risk factors and recognizing characteristic skin manifestations indicative of EH, proves valuable for accurate diagnosis. EH can lead to fatal outcomes if it disseminates throughout the body, underscoring the importance of early detection upon the appearance of initial symptoms and prompt initiation of treatment.

## References

[1] Torres T, Ferreira EO, Gonçalo M, Mendes-Bastos P, Selores M, Filipe P (2019). Update on atopic dermatitis. Acta Med Port.

[2] Traidl S, Roesner L, Zeitvogel J, Werfel T (2021). Eczema herpeticum in atopic dermatitis. Allergy.

[3] Werfel T, Allam JP, Biedermann T (2016). Cellular and molecular immunologic mechanisms in patients with atopic dermatitis. J Allergy Clin Immunol.

[4] Wollenberg A, Wetzel S, Burgdorf WH, Haas J (2003). Viral infections in atopic dermatitis: pathogenic aspects and clinical management. J Allergy Clin Immunol.

[5] Wetzel S, Wollenberg A (2004). Eczema herpeticatum (Article in German). Hautarzt.

[6] Beck LA, Boguniewicz M, Hata T (2009). Phenotype of atopic dermatitis subjects with a history of eczema herpeticum. J Allergy Clin Immunol.

[7] Luca NJ, Lara-Corrales I, Pope E (2012). Eczema herpeticum in children: clinical features and factors predictive of hospitalization. J Pediatr.

[8] Bussmann C, Peng WM, Bieber T, Novak N (2008). Molecular pathogenesis and clinical implications of eczema herpeticum. Expert Rev Mol Med.

[9] Wollenberg A (2012). Eczema herpeticum. Chem Immunol Allergy.

[10] Novak N, Peng WM (2005). Dancing with the enemy: the interplay of herpes simplex virus with dendritic cells. Clin Exp Immunol.

[11] Cunningham AL, Diefenbach RJ, Miranda-Saksena M, Bosnjak L, Kim M, Jones C, Douglas MW (2006). The cycle of human herpes simplex virus infection: virus transport and immune control. J Infect Dis.

[12] Gogou M, Douma S, Haidopoulou K, Giannopoulos A (2018). Herpeticum-like rash in a child with atopic dermatitis: early clinical suspicion is valuable. Sudan J Paediatr.

[13] Besh L, Matsyura O, Besh O (2020). Eczema herpeticum in an infant - a case report. Pediatr Med Rodz.

[14] Aronson PL, Yan AC, Mittal MK, Mohamad Z, Shah SS (2011). Delayed acyclovir and outcomes of children hospitalized with eczema herpeticum. Pediatrics.

[15] Almoalem M, AlAlhareth I, Alomer H (2022). Extensive eczema herpeticum in a previously well child. Int J Emerg Med.

[16] Liaw FY, Huang CF, Hsueh JT, Chiang CP (2012). Eczema herpeticum: a medical emergency. Can Fam Physician.

